# Does obesity and varying body mass index affect the clinical outcomes and safety of biportal endoscopic lumbar decompression? A comparative cohort study

**DOI:** 10.1007/s00701-024-06110-1

**Published:** 2024-06-03

**Authors:** Thomas E. Olson, Alexander Upfill-Brown, Babapelumi Adejuyigbe, Nitin Bhatia, Yu-Po Lee, Sohaib Hashmi, Hao-Hua Wu, Hansen Bow, Cheol Wung Park, Dong Hwa Heo, Don Young Park

**Affiliations:** 1 UCLA Department of Orthopaedic Surgery, David Geffen School of Medicine at UCLA, Los Angeles, CA USA; 2 UC Irvine Department of Orthopaedic Surgery, UC Irvine School of Medicine, 101 The City Drive South, Pavillion III, Building 29A, Orange, CA 92868 USA; 3 Department of Neurosurgery, Woori Hospital, Seoul, South Korea; 4 Department of Neurosurgery, Harrison Spinartus Hospital Chungdam, Seoul, South Korea

**Keywords:** Endoscopic Spine Surgery, Biportal Spinal Endoscopy, Lumbar Discectomy, Lumbar Decompression, Obesity, Comorbidities

## Abstract

**Background:**

Endoscopic spine surgery has recently grown in popularity due to the potential benefits of reduced pain and faster recovery time as compared to open surgery. Biportal spinal endoscopy has been successfully applied to lumbar disc herniations and lumbar spinal stenosis. Obesity is associated with increased risk of complications in spine surgery. Few prior studies have investigated the impact of obesity and associated medical comorbidities with biportal spinal endoscopy.

**Methods:**

This study was a prospectively collected, retrospectively analyzed comparative cohort design. Patients were divided into cohorts of normal body weight (Bone Mass Index (BMI)18.0–24.9), overweight (BMI 25.0–29.9) and obese (BMI > 30.0) as defined by the World Health Organization (WHO). Patients underwent biportal spinal endoscopy by a single surgeon at a single institution for treatment of lumbar disc herniations and lumbar spinal stenosis. Demographic data, surgical complications, and patient-reported outcomes were analyzed. Statistics were calculated amongst treatment groups using analysis of variance and chi square where appropriate. Statistical significance was determined as p < 0.05.

**Results:**

Eighty-four patients were followed. 26 (30.1%) were normal BMI, 35 (41.7%) were overweight and 23 (27.4%) were obese. Patients with increasing BMI had correspondingly greater American Society of Anesthesiologist (ASA) scores. There were no significant differences in VAS Back, VAS Leg, and ODI scores, or postoperative complications among the cohorts. There were no cases of surgical site infections in the cohort. All cohorts demonstrated significant improvement up to 1 year postoperatively.

**Conclusions:**

This study demonstrates that obesity is not a risk factor for increased perioperative complications with biportal spinal endoscopy and has similar clinical outcomes and safety profile as compared to patients with normal BMI. Biportal spinal endoscopy is a promising alternative to traditional techniques to treat common lumbar pathology.

**Supplementary Information:**

The online version contains supplementary material available at 10.1007/s00701-024-06110-1.

## Introduction

As the United States grapples with a growing obesity epidemic, marked by a 20% increase in adult body mass index (BMI) and obesity rates over the last decade, the surgical community faces critical challenges [16, 25, 30]. Elevated BMI has been strongly linked with an increased risk of complications across a spectrum of surgical interventions, and spine surgery is no exception. These complications can range from infection, prolonged hospital stays, and readmission rates to more severe outcomes such as nerve injury, post-operative bleeding, venous thromboembolism, and mortality [1, 11, 12, 20–22, 32, 38].

Minimally invasive surgical (MIS) techniques have emerged as promising alternatives to traditional open surgery in the approach towards reducing some of the aforementioned complications [7, 11]. Biportal spinal endoscopy, in particular, has recently developed into an effective ultra-MIS technique for treating common lumbar pathologies such as lumbar disc herniations and lumbar stenosis [14, 17, 19, 23]. This technique utilizes water-based irrigation, visualization with an endoscope through a viewing portal, and introduction of surgical instruments through a separate working portal to access spinal anatomy. Compared with traditional MIS techniques, this results in smaller incisions with less soft tissue dissection and has been shown to result in improved pain, increased mobilization, and shorter length of stay [9, 33, 35, 40]. Multiple clinical studies, systematic reviews, and meta-analyses of biportal endoscopic techniques have demonstrated excellent clinical results with low complication rates [18, 28, 36, 39].

Given the relevance of potential benefits for the at-risk obese population, only a few studies have examined endoscopic techniques in obese patients with promising results [3–5, 10, 26, 34]. While biportal techniques show promise for greater surgical precision and potentially fewer complications, there is a lack of clinical studies in the literature examining the impact of obesity in biportal spinal endoscopy with only 2 prior studies investigating biportal discectomies but no prior study investigating biportal decompression for stenosis [10, 34].

This present study investigates the early clinical outcomes of biportal endoscopic decompression for lumbar disc herniations and lumbar spinal stenosis across various patient-specific factors such as BMI and medical comorbidity burden. Our aim of this study was to determine the clinical results with biportal spinal endoscopy in patients with various BMI and medical comorbidities, particularly with obese patients. We hypothesize that obesity is not a risk factor for worse clinical outcomes or greater complications with biportal spinal endoscopy as compared to normal BMI individuals. We also describe a novel radiographic method to preoperatively plan the portal incision sites with MRI imaging, which is particularly important since obesity is associated with increased soft tissue from skin to spine.

## Methods

Consecutive patients undergoing biportal spinal endoscopy by a single surgeon with an expertise in biportal spinal endoscopy were included in this study. All procedures were performed at a tertiary care university hospital system in the United States. The study was a prospectively collected, retrospectively analyzed and our study design was retrospective and IRB-approved (IRB#22–001674). Thus far, the research dataset produced 2 prior publications using the data collected [37, 42]. Inclusion criteria consisted of all primary biportal spinal endoscopy procedures in the lumbar spine starting with the initiation of the study period in October 2021 through February 2023 for the diagnosis of lumbar disc herniation, lumbar stenosis, and lumbar synovial facet cyst causing stenosis requiring surgery for lumbar radiculopathy. Exclusion criteria included any revision surgery and any surgery in the context of spinal instability, infection, tumor, or trauma. Patients were divided by body mass index (BMI) classification of normal body weight (BMI 18.0–24.9), overweight (BMI 25.0–29.9) and obese (BMI > 30.0) as defined by the World Health Organization (WHO) [31].

**Fig. 1:**
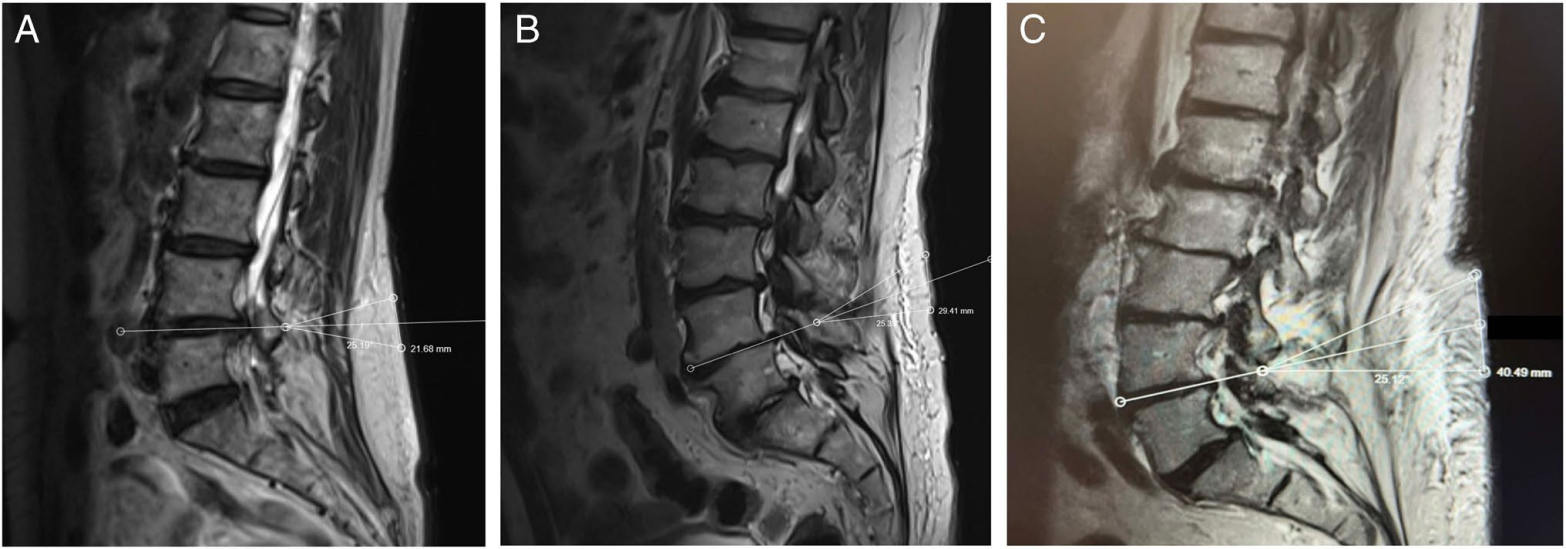
Method depicted in preoperative planning based on the T2-weighted sagittal image showing the target level and lamina of interest. The disc space line is first drawn and the 2 lines above and below the disc space line are drawn from the skin level to the target lamina that are equidistant to the disc space line. The lines converge to the target lamina at an angle of approximately 25 degrees. The distance between the 2 lines is then measured to determine the distances between the portals. Portals are typically 2 cm apart for patients with BMI of 20–25 (**A**), 3 cm apart for BMI 25–30 (**B**), > 4 cm apart for BMI > 30 (**C**)

The standard biportal spinal endoscopy technique was performed as previously described in prior publications, using standard arthroscopic equipment, a radiofrequency machine and probes, high speed bur, bone cutting shavers, standard spinal instruments (e.g. pituitaries, Kerrisons, etc.), and C-arm fluoroscopy [14, 17, 19, 23]. Preoperative planning was required for all cases based on the sagittal T2-weighted MRI sequences to ensure that the endoscope and surgical instruments triangulate correctly to the target lamina (Fig. 1). With increased soft tissue from the skin to the spine, the portal incisions must be made farther apart, otherwise the endoscope and surgical instruments will conflict with each other and the surgery would be difficult to perform.

**Fig. 2:**
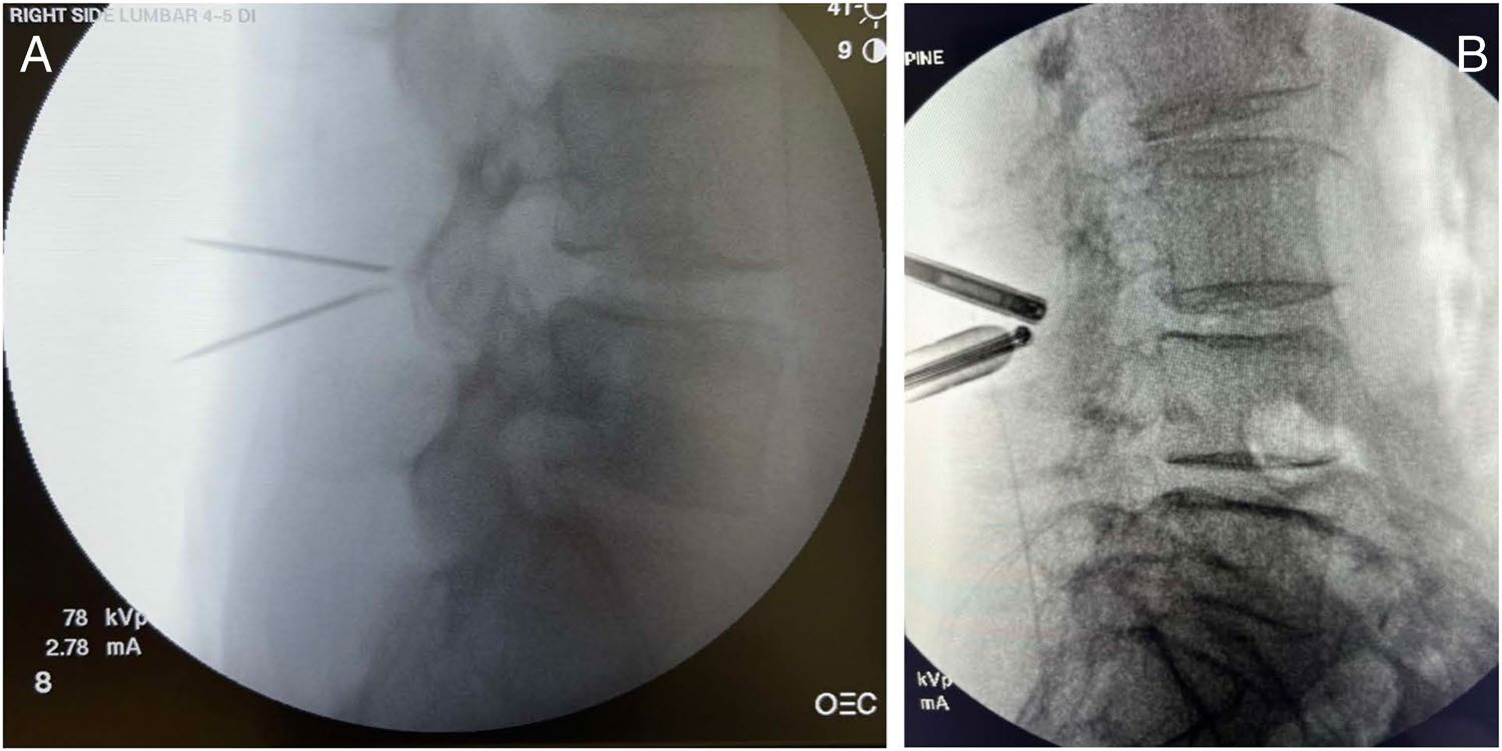
Lateral fluoroscopic image depicted showing the two spinal needles converging to the target lamina based on the preoperative planning (**A**). These spinal needles signified the trajectories of the viewing and working portals for the biportal endoscopic procedure. After making incisions, the endoscopic trochar and surgical instruments are then placed and triangulated over the target lamina in the same trajectories (**B**)

**Fig. 3:**
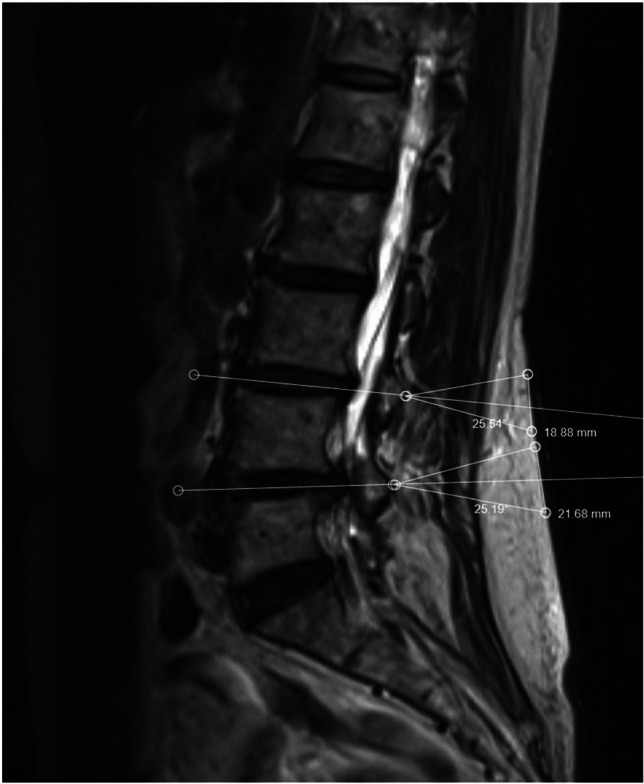
Two level method depicted in preoperative planning based on the T2-weighted sagittal image showing the target level and lamina of interest. Incisions that were within 4–5 mm of each other were typically combined and used for the second level, for a total of 3 incisions

A line was drawn through the disc space and projected to the skin level if visible. Two lines were drawn above and below the disc space line equidistant to the disc space line, converging from the skin level to the target lamina. An approximately 25-degree angle was utilized between the lines. These lines represented the trajectories of the viewing and working portals and measurements were obtained between the lines to determine the distance required between the portals for preoperative planning. In general, using this technique, normal BMI required approximately 2 cm distance between the portals (Fig. 1A), overweight BMI required 3 cm (Fig. 1B), and obese BMI required > 4 cm (Figs. 1C). These measurements were then used to determine the incision sites during surgery. The target disc space was determined using lateral fluoroscopy and 2 spinal needles were used to determine the portal trajectories (Fig. 2A,B). The spinal needles were placed using the distances determined by the preoperative planning method described above. For all cases regardless of BMI, the viewing portal incision measured approximately 6–7 mm and the working portal incision measured 10–12 mm in length. No changes in the incision length or any specialized surgical steps or retractors were necessary during surgery for all cases. For two level surgeries, additional incisions were necessary for the placement of the endoscope and surgical instruments after completion of the first level. This was planned preoperatively by the method described above (Fig. 3).

Depending on the pathology, lumbar discectomy, lumbar laminotomy and decompression were performed utilizing the biportal endoscopic technique in the standard fashion [14, 17, 19, 23]. All cases in all three cohorts had post-operative drains placed at the end of surgery as part of the study protocol. The drains were removed immediately prior to discharge home for outpatient surgeries or on post-operative day one for inpatient surgeries.

All patients completed previously validated patient-reported outcome (PRO) measures consisting of Visual Analog Scores (VAS) for back and leg pain and the Oswestry Low Back Disability Index (ODI) at the initial preoperative visit and all subsequent postoperative visits [13]. All post-operative complications including neurological changes such as recurrent leg pain, numbness, tingling, and motor weakness at all points were recorded in the follow-up period. The follow-up intervals were six weeks, three months, six months, and one year after surgery. Telehealth visits were performed with all patients who had missed any follow-up visits after the procedure. A secure Health Insurance Portability and Accountability Act (HIPAA) compliant institutional database was utilized to collect all demographic, perioperative data, complications, patient-reported outcomes, American Society of Anesthesiologists (ASA) and Charlson Comorbidity Index (CCI) scores.

### Statistical methods

The study’s primary outcomes included changes in PROs and post-operative complications. All perioperative data, demographics, ASA and CCI scores were compared between the cohorts. Visual inspection and the Shapiro–Wilk test was used to assess for normality of continuous variables, with p < 0.10 for the latter indicative of non-normally distributed data (Supplemental Table 1). For skewed, nonparametric distributions, continuous variables are presented as median and interquartile range (IQR) and analyzed using the Kruskal–Wallis test. Chi-squared tests were used for categorical analyses in which the expected categorical outcome was greater than five, and Fisher’s exact test was instead used when the expected value for any given categorical outcome was less than five. Statistical analyses were performed using R 3.6.0 (R Foundation for Statistical Computing, Vienna, Austria).

## Results

### Patient demographics

84 patients were identified as having met inclusion criteria. Of them, 26 (30.1%) were within normal body weight limits [BMI 18.0–24.9], 35 (41.7%) were classified as overweight [BMI 25.0–29.9] and 23 (27.4%) were obese [BMI > 30.0]. There was no significant difference in mean follow up time (7.5 months, p = 0.4550), age of patients at the time of surgery (60.0 years old, Table 1, p = 0.5067), or number of surgeries performed in the outpatient setting (69%, Table 1, p = 0.2935) between various BMI cohorts. Significantly fewer female patients were found within the overweight and obese patient groups (Table 1, p = 0.0192). There was a significant association between increased ASA scores and increasing BMI (Table 1, p < 0.0001), although there was no corollary association with CCI (Table 1, p = 0.3692).

**Table 1:** Demographic characteristics of patients undergoing biportal spinal endoscopy

Variable	Statistic	Normal Weight	Overweight	Obese	Total	P-Value
		(n = 26)	(n = 35)	(n = 23)	(n = 84)	
Female^*^	N (%)	13 (50%)	6 (17%)	6 (26%)	25 (30%)	0.0192
Age (yrs)	Mean (SD)	56.9 (19.2)	62.3 (15.4)	60.0 (14.5)	60.0 (16.4)	0.5067
BMI (kg/m^2^)	Mean (SD)	22.6 (1.3)	27.4 (1.4)	33.6 (2.6)	27.6 (4.6)	< 0.0001
ASA score	Mean (SD)	1.8 (0.7)	2.4 (0.5)	2.7 (0.5)	2.3 (0.6)	< 0.0001
Charlson Comorbidity Index	Mean (SD)	1.8 (1.9)	2.3 (2.0)	2.5 (1.8)	2.2 (1.9)	0.3692
Outpatient Procedure^*^	N (%)	19 (73%)	21 (60%)	18 (78%)	58 (69%)	0.2935
Surgical Duration (min)	Mean (SD)	111 (43)	133 (48)	128 (47)	125 (47)	0.1329
Total Drain Output (mL)	Median (IQR)	35 (25–85)	48 (25–86)	30 (7–61)	35 (25–80)	0.3192

^*^Expected categorical outcomes greater than five, analysis via chi square test

### Surgical distribution and outcomes

The cases performed included 67 single-level and 17 two-level decompressive procedures spanning from L1 to S1, with no statistically significant differences between the three cohorts in the number of levels (Table 2, p = 0.5824) or the distribution of the specific levels addressed (Table 2, p = 0.4802). The most frequently addressed levels were L4-L5, followed by L5-S1 and L3-L4. There were significantly more disc herniations and consequently discectomies (62%, Table 2, p = 0.0257) in those of normal weight as compared to overweight and obese patients, who had more stenosis diagnoses with subsequent decompression (66–74%, Table 2, p = 0.0576). Amongst all patients, the median surgical duration was 125 min with no observed difference between study groups (Table 1, p = 0.1693). Similarly, there was no difference in cumulative drain output (median 35 mL, Table 1, p = 0.3192).

**Table 2:** Surgical features of patients undergoing biportal spinal endoscopy

Variable		Normal Weight	Overweight	Obese	Total	P-value
		(n = 26)	(n = 35)	(n = 23)	n = 84	
Number of Levels^†^	1 Level	22 (85%)	26 (74%)	19 (83%)	67 (80%)	0.5824
	2 Levels	4 (15%)	9(26%)	4(17%)	17 (20%)	
Levels Addressed^*†^	L1-2	0 (0%)	2 (5%)	0 (0%)	2 (2%)	0.4802
	L2-3	1 (3%)	4 (9%)	3 (11%)	8 (8%)	
	L3-4	3 (10%)	10 (23%)	7 (26%)	20 (20%)	
	L4-5	17 (57%)	20 (45%)	13 (48%)	50 (50%)	
	L5-S1	9 (30%)	8 (20%)	4 (15%)	21 (21%)	
Primary Diagnosis^‡^	Stenosis	10 (38%)	23 (66%)	17 (74%)	50 (60%)	0.0257
	Disc herniation	16 (62%)	12 (34%)	6 (26%)	34 (40%)	
Primary Procedure^‡^	Discectomy	16 (62%)	12 (34%)	6 (26%)	34 (40%)	0.0257
	Lami/Decomp	10 (38%)	23 (66%)	17 (74%)	50 (60%)	

^*^Total number of levels addressed for normal weight, overweight and obese patients are 30, 44, and 27, respectively. 101 levels were addressed among all patients

^†^Expected categorical outcomes less than five, analysis via Fisher exact test

^‡^Expected categorical outcomes greater than five, analysis via chi square test

### Postoperative complications

No significant differences were detected with postoperative complications such as transient postoperative radiculitis, postoperative weakness, wound complications, or reherniation during the postoperative follow-up period between the three cohorts (Table 3, p = 0.7680, 0.7390, 0.5833, 0.1658, respectively). For this study, postoperative radiculitis was defined as any occurrence of radiating leg pain, numbness, or tingling that occurred in the postoperative period. All cases of transient postoperative radiculitis were transient and resolved by the six week point post-operatively with conservative treatment using oral anti-inflammatory medications and/or oral steroids as needed. There were two cases of postoperative weakness with grade 4/5 EHL weakness that improved with physical therapy and rehabilitation. There were no revision surgeries performed after the index surgery related to any postoperative complication.

**Table 3:** Complications following biportal endoscopic lumbar surgery

Complication	Normal Weight	Overweight	Obese	Total	P-value
	(n = 26)	(n = 35)	(n = 23)	(n = 84)	
Postoperative Radiculitis^*^	5 (19%)	5 (14%)	5 (22%)	15 (18%)	0.7680
Postoperative Weakness^*^	1 (4%)	1 (3%)	0 (0%)	2 (2%)	0.7390
Wound Drainage^*^	1 (4%)	0 (0%)	0 (0%)	1 (1%)	0.5833
Reherniation^*^	2 (8%)	0 (0%)	0 (0%)	2 (2%)	0.1658

^*^Expected categorical outcomes less than five, analysis via Fisher exact test

### Patient reported outcomes

**Fig. 4:**
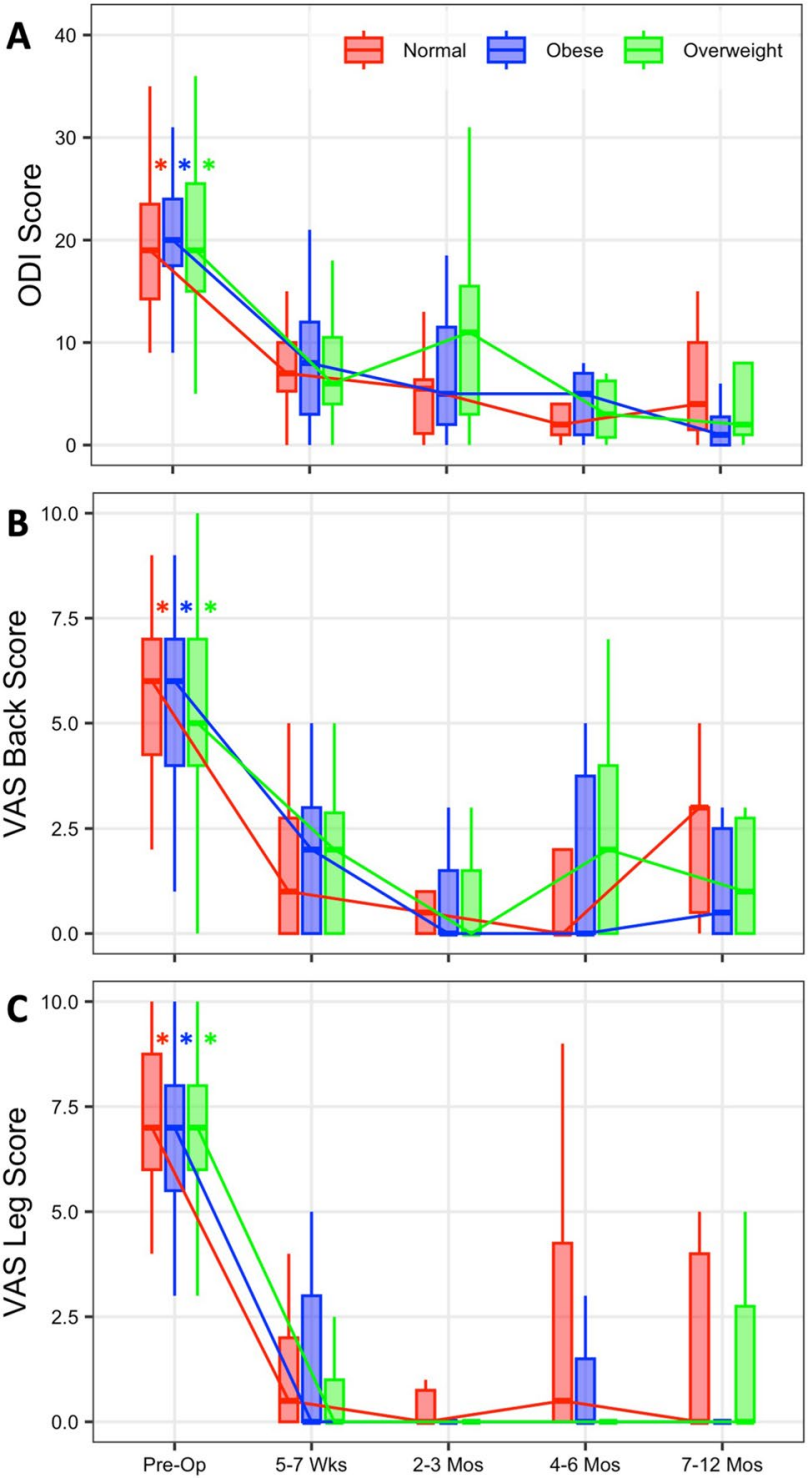
Trend in patient reported outcomes across different peri-operative time points for (**A**) ODI score, (**B**) VAS Back Score, and (**C**) VAS Leg Score. Figures represent box and whisker plots with median and inter-quartile range highlighted in boxes and whiskers at 1.5 range coefficient, comprising approximately 99% of the maximum data range. Significance denoted in asterisks, representing difference in preoperative values from all postoperative time points. There were no significant differences between groups at all measured intervals

**Fig. 5:**
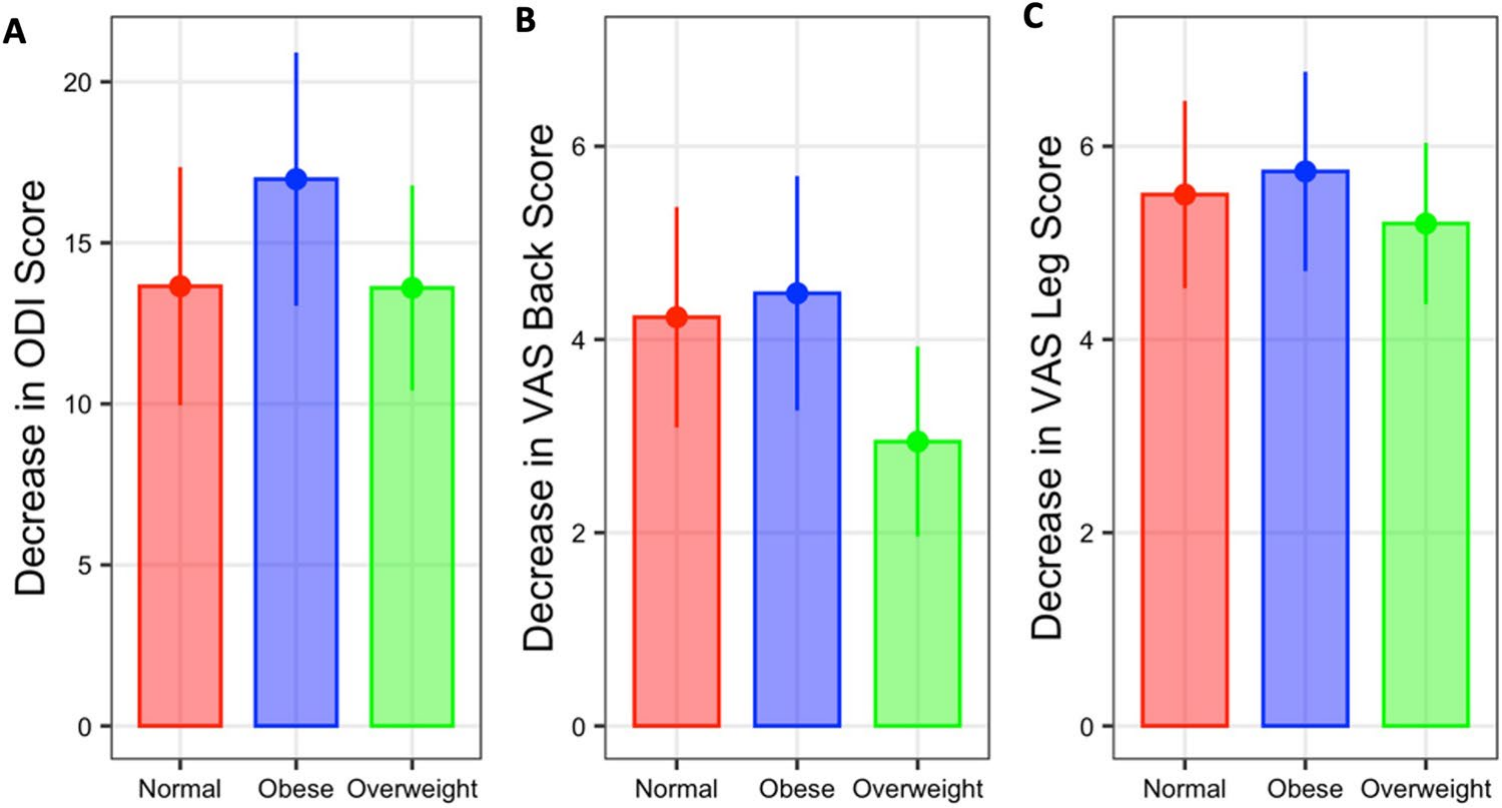
Absolute difference in patient reported outcomes between pre-op and most recent follow up for (**A**) ODI score, (**B**) VAS Back score and (**C**) VAS Leg score. Figures represent mean and 95% confidence interval. No significant differences were observed between groups

PROs improved significantly from pre-op values in all groups and at each measured follow up interval (Fig. 4, p < 0.0001). No statistically significant differences were observed between the cohorts at preoperative baseline or at any of the measured follow up intervals for ODI, VAS back, or VAS leg values (Fig. 4, Supplemental Table 2, p = 0.1803–0.9604). At final follow up, patients in the normal weight cohort had improved ODI score by an average of 14 points (Fig. 5A, p < 0.0001), VAS back by an average of 4.2 points (Fig. 5B, p < 0.0001), and VAS leg scores by an average of 5.5 points (Fig. 5C, p < 0.0001). Patients in the overweight cohort improved final ODI scores by an average of 13 points (Fig. 5A, p < 0.0001), mean VAS back by 3.0 points, (Fig. 5B, p < 0.0001), and mean VAS leg scores by 5.2 points (Fig. 5C, p < 0.0001). Patients in the obese cohort demonstrated improvement in ODI score by an average of 17 points at final follow up (Fig. 5A, p < 0.0001), as well as 4.5 points in VAS back scores (Fig. 5B, p < 0.0001) and 5.7 points in VAS leg scores (Fig. 5C, p < 0.0001). Although the improvement in ODI scores was the greatest in the obese group, the overall amount of improvement in PROs between BMI cohorts was not statistically different (Fig. 5, p = 0.2059–0.8587).

## Discussion

The ongoing obesity epidemic in the United States has brought into focus the implications of obesity and medical comorbidities on surgical outcomes, particularly in spine surgery. Our study sought to investigate the clinical outcomes of biportal spinal endoscopy in patients with varying BMI and comorbidity profiles to identify potential risk factors. Our results support our hypothesis that obesity is not a significant risk factor in worse clinical outcomes or increased risk for complications in biportal spinal endoscopy performed for common lumbar pathologies, such as lumbar disc herniations and lumbar stenosis. To our knowledge, this is the first clinical study investigating biportal endoscopic decompression for the treatment of lumbar disc herniation and lumbar spinal stenosis with direct comparison of obese, overweight, and normal patients with their associated medical comorbidities. In addition, we describe a novel radiographic method to preoperatively determine the approximate locations and distances between the portal incision sites based on T2-weighted sagittal MRI images. Based on our results, the preoperative planning methods proved useful to performing the biportal endoscopy procedure successfully across all BMI cohorts. The procedure can be successfully performed in obese patients without any additional specialized surgical steps, intraoperative navigation, or specialized retractors with the same size incisions as normal BMI patients. This may help reduce surgical time, cost, and complications, especially surgical site infections as compared to open surgery.

In a meta-analysis by Goyal et al., obese patients undergoing open treatment of degenerative lumbar pathologies demonstrated an overall odds ratio (OR) of 1.34 for surgical and postoperative complications, as well as an OR of 1.40 for reoperation as compared to non-obese counterparts [15]. These differences from the non-obese population, however, were negated for patients undergoing minimally invasive procedures. Further analysis between specific minimally invasive techniques amongst obese patients have been more recently explored in a retrospective comparative study by Choi et al., in which a notable trend towards wound complications (7% v. 0%), reherniation (42% v. 23%), and reoperation (19% v. 3%) was observed in microscopic decompression as compared to biportal endoscopic techniques [10]. However, the findings were statistically insignificant due to the small sample size. In addition, Choi et al. did not compare the results of obese patients with non-obese patients like our present study. The only other biportal endoscopic study investigating obesity was by Park, et al., who compared 29 obese and 86 non-obese patients in a multi-center retrospective design and demonstrated no difference in PROs or complications, similarly to our study [34]. Park, et al. only included biportal discectomy cases, whereas our present study included both biportal discectomies and decompressions for lumbar stenosis and further differentiated the study population into normal, overweight, and obese categories defined by the WHO [37]. While early in the experience of this emerging technique, it is evident that there is a potential benefit of biportal endoscopic techniques in this at-risk obese population.

Patients in our study were categorized according to WHO-defined BMI cohorts: normal weight, overweight, and obese. Importantly, despite increasing ASA scores, more medical comorbidities, and potential risk associated with greater BMI, no significant differences were observed across cohorts in terms of surgical complications, such as transient postoperative radiculitis, weakness, wound complications, infections, or recurrence. This finding is consistent with the recently published retrospective review by Bergquist et al., who observed no difference in perioperative complications between obese and non-obese patients [5]. Furthermore, previously published association of increased operative times with obese patients in traditional open procedures, such as reported in the meta-analysis by Cao et al., were not observed in our study [6].

There were few overall complications in this study. The most common postoperative complication observed in this patient study was transient radiculitis, which was present in all BMI cohorts. This issue may be related to the postoperative inflammation of the neural elements that may occur from retraction or manipulation, rather than obesity or BMI. In all cases of postoperative radiculitis, patients initially reported resolution of their preoperative radicular symptoms after surgery. After 2–3 days after surgery, patients who developed postoperative radiculitis would describe recurrence of their radicular symptoms but to a lesser degree than preoperatively. Postoperative MRIs were not routinely obtained in these cases as they all resolved with medical management such as NSAIDs and/or a short oral steroid course with methylprednisolone. This led us to believe that the postoperative radiculitis was likely due to nerve root inflammation that developed 2–3 days after surgery and not due to other etiology such as nerve injury from persistent neural compression. There were no instances of excessive manipulation of the nerve root during surgery, which would more likely cause immediate postoperative symptomatology and/or neurological deficits. While not routinely reported in the literature as a complication due to its transient nature and likely under-reported, a recent meta-analysis by Park et al. demonstrated a reported rate of nerve root injury and transient radiculitis at 0.24% within the published biportal endoscopic lumbar complications [36]. Although occurring at a higher rate within our study, all instances of transient postoperative radiculitis in this study resolved by the six-week follow-up with conservative management.

There was one case of postoperative weakness in each of the normal and overweight cohorts. Postoperative MRIs were obtained in both of these cases, which demonstrated a small epidural hematoma that was treated nonsurgically, and the weakness improved over time with physical therapy and rehabilitation. Studies performing routine post-operative MRIs have shown epidural hematoma rates of 23.6–24.7% following biportal decompression surgery [24, 44]. Of note, the overwhelming majority of these epidural hematomas are asymptomatic, with only 1.2–5.1% of patients undergoing revision surgery for hematoma evacuation. Previous studies, such as that by Snopko et al., have shown in traditional open approaches, obesity can be an independent risk factor for symptomatic epidural hematoma [41]. However, our results did not yield any increased risk of this complication due to obesity, which may be due to endoscopic techniques requiring excellent hemostasis for intra-operative visualization, in addition to less bony resection and creation of dead space as compared to open surgery, thereby providing a protective effect in an at-risk population [2, 5, 26, 34, 43].

We did not find any significant difference in PROs amongst the various BMI groups. Each cohort exhibited significant improvements in VAS Back, VAS Leg, and ODI scores as compared to preoperative scores, which were statistically indistinguishable from one another and similar to the values reported in a recent meta-analysis of the general population by Park et al.[36] These improvements were well beyond the reported minimum clinically important difference (MCID) for ODI, VAS leg, and VAS back scores of 12.8, 1.6, and 1.2, respectively, as reported by Copay et al.[6] More recently, the value of a rigid MCID has been questioned, and a range allowing for calibration of specificity and sensitivity has been introduced. The PROs demonstrated by patients in this study remain exceedingly greater than the published MCID range for VAS scores of 2.5 to 3.5 published by Lewandrowski et al. and fall directly within the suggested MCID range of 14 to 17 for ODI [27]. Furthermore, there was no difference between the preoperative PROs of the various cohorts, indicating that the characteristics of the cohorts were quite similar. Our study’s results challenge the often-held belief that higher BMI and obesity inherently leads to unfavorable clinical outcomes, at least within the context of biportal spinal endoscopy. Our results correlate well with the existing published literature that biportal spinal endoscopy is indeed safe and effective in treating common lumbar pathologies, now including overweight and obese patients with proper surgical indications [3, 5, 26, 29, 34].

Regarding comorbidities, our data demonstrated a significant increase in the ASA scores with increased BMI. Although there was an increasing trend in mean CCI scores with increasing BMI, no significant differences were observed between the cohorts with CCI. CCI incorporates age and multiple medical diagnoses including cardiac disease, peripheral vascular disease, stroke, dementia, COPD, diabetes, kidney disease, and cancers to calculate a score and estimate 10-year survival [8]. CCI may be a more specific assessment of the patient’s comorbidities than ASA scores. Despite these differences, there were no increased risk of complications with obesity as compared to the normal and overweight cohorts. Our results demonstrate that obesity itself is not a risk factor for biportal spinal endoscopy, but careful consideration should be made at the discretion of the surgeon to properly indicate obese patients for surgery in the context of their other comorbidities.

The limitations of this study include the small sample size and short duration of follow-up, which may influence the results of the study. This study’s follow-up period may not sufficiently capture disc herniation recurrences, which may take a longer time period to occur. Subtle differences in rare adverse outcomes may be brought out with greater sample sizes and longer follow-up duration. However, our results corroborate well with the published studies in the literature. Another limitation of this study was the inclusion of both biportal discectomies and biportal decompressions, as well as 1-level and 2-level surgeries, making the study size larger but also heterogenous. We wanted to include biportal decompressions for lumbar spinal stenosis since no prior study has investigated the impact of BMI in this patient population.

Regarding data collection, the study design incorporated the prospective collection of patient data with retrospective analysis, which can also introduce selection bias in the study. In addition, patients may more favorably respond to completing PRO questionnaires due to novelty of biportal spinal endoscopy, potentially leading to response bias. The study was designed at the inception to investigate the clinical results of biportal spinal endoscopy with patients of varying BMI, and all clinical outcomes with operative and complication data were collected at the outset prospectively by the study staff. However, certain elements of the study data, such as the ASA score and CCI were collected from the electronic medical record retrospectively and the data analysis for the study was completed retrospectively, which does limit the study’s results and conclusions.

## Conclusions

This comparative cohort study demonstrated that biportal spinal endoscopy for lumbar decompression was both safe and effective when performed in appropriately selected patients of varying BMI with no difference in outcomes or complications between normal, overweight, and obese patients. Improved clinical outcomes with low complication rates were observed in all cohorts, demonstrating clinical success with the technique regardless of BMI. We demonstrated that properly selected overweight and obese patients can safely undergo biportal endoscopic decompression without increased risk. Overall, these findings in this study are consistent with the existing literature and lay the groundwork for larger well-designed, multi-center prospective studies with longer follow-up that can further elucidate the interplay between BMI, medical comorbidities, clinical outcomes, and complications in biportal spinal endoscopy.

## Supplementary Information

Below is the link to the electronic supplementary material.

Supplementary file1 (DOCX 14 KB)

## Data Availability

De-identified study data will be available upon request from the corresponding author.

## Abbreviations

BMI: Bone Mass Index
MIS: Minimally Invasive Surgery
WHO: World Health Organization
ASA: American Society of Anesthesiologist
PRO: Patient Reported Outcome
VAS: Visual Analog Score
ODI: Oswestry Low Back Disability Index
CCI: Charlson Comorbidity Index
IQR: Interquartile Range
