# Supplementary material for: Does obesity and varying body mass index affect the clinical outcomes and safety of biportal endoscopic lumbar decompression? A comparative cohort study
Source: Acta Neurochir (Wien). 2024 Jun 3;166(1):246. doi: 10.1007/s00701-024-06110-1 (PMC11147858; doi:10.1007/s00701-024-06110-1)
Supplement: Supplementary file 1 — Supplementary file1 (DOCX 14 KB) [file 701_2024_6110_MOESM1_ESM.docx]

Supplemental Table 1 – Shapiro-Wilks Tests for Normal Distribution

| **Variable** | **P Value** |
| --- | --- |
| Age | 0.0007 |
| Follow Up Duration | < 0.0001 |
| BMI | < 0.0001 |
| ASA | 0.0380 |
| CCI | <0.0001 |
| Surgical Duration | <0.0001 |
| EBL | <0.0001 |
| Total Drain Output | <0.0001 |

Supplemental Table 2 – Kruskall-Wallace P Values for PRO time intervals

| **Time Interval** | **ODI** | **VAS Back** | **VAS Leg** |
| --- | --- | --- | --- |
| Pre Op | 0.7622 | 0.3803 | 0.8756 |
| 2 Weeks | 0.2019 | 0.2019 | 0.1353 |
| 5-7 Weeks | 0.9301 | 0.8251 | 0.7415 |
| 2-3 Months | 0.4847 | 0.9736 | 0.2034 |
| 7-12 months | 0.8352 | 0.6045 | 0.4124 |
| 4-6 Months | 0.3709 | 0.5776 | 0.4132 |
